# Cerebral perfusion in sepsis-associated delirium

**DOI:** 10.1186/cc6891

**Published:** 2008-05-05

**Authors:** David Pfister, Martin Siegemund, Salome Dell-Kuster, Peter Smielewski, Stephan Rüegg, Stephan P Strebel, Stephan CU Marsch, Hans Pargger, Luzius A Steiner

**Affiliations:** 1 Department of Anaesthesia, Operative Intensive Care Unit, University Hospital Basel, Spitalstrasse 21, CH-4031 Basel, Switzerland; 2 Academic Neurosurgery, Addenbrooke's Hospital, Cambridge University Hospitals NHS Foundation Trust, Hills Road, Cambridge CB2 0QQ, UK; 3 Department of Neurology, University Hospital Basel, Petersgraben 4, CH-4031 Basel, Switzerland; 4 Medical Intensive Care Unit, University Hospital Basel, Petersgraben 4, CH-4031 Basel, Switzerland

## Abstract

**Introduction:**

The pathophysiology of sepsis-associated delirium is not completely understood and the data on cerebral perfusion in sepsis are conflicting. We tested the hypothesis that cerebral perfusion and selected serum markers of inflammation and delirium differ in septic patients with and without sepsis-associated delirium.

**Methods:**

We investigated 23 adult patients with sepsis, severe sepsis, or septic shock with an extracranial focus of infection and no history of intracranial pathology. Patients were investigated after stabilisation within 48 hours after admission to the intensive care unit. Sepsis-associated delirium was diagnosed using the confusion assessment method for the intensive care unit. Mean arterial pressure (MAP), blood flow velocity (FV) in the middle cerebral artery using transcranial Doppler, and cerebral tissue oxygenation using near-infrared spectroscopy were monitored for 1 hour. An index of cerebrovascular autoregulation was calculated from MAP and FV data. C-reactive protein (CRP), interleukin-6 (IL-6), S-100β, and cortisol were measured during each data acquisition.

**Results:**

Data from 16 patients, of whom 12 had sepsis-associated delirium, were analysed. There were no significant correlations or associations between MAP, cerebral blood FV, or tissue oxygenation and sepsis-associated delirium. However, we found a significant association between sepsis-associated delirium and disturbed autoregulation (*P *= 0.015). IL-6 did not differ between patients with and without sepsis-associated delirium, but we found a significant association between elevated CRP (*P *= 0.008), S-100β (*P *= 0.029), and cortisol (*P *= 0.011) and sepsis-associated delirium. Elevated CRP was significantly correlated with disturbed autoregulation (Spearman rho = 0.62, *P *= 0.010).

**Conclusion:**

In this small group of patients, cerebral perfusion assessed with transcranial Doppler and near-infrared spectroscopy did not differ between patients with and without sepsis-associated delirium. However, the state of autoregulation differed between the two groups. This may be due to inflammation impeding cerebrovascular endothelial function. Further investigations defining the role of S-100β and cortisol in the diagnosis of sepsis-associated delirium are warranted.

**Trial registration:**

ClinicalTrials.gov NCT00410111.

## Introduction

Sepsis-associated delirium is one of the most common causes of delirium in intensive care units [1]. Sepsis-associated delirium is not simply an unpleasant confusion or obtundation of a patient with sepsis, but a relevant and often severe organ dysfunction that is reflected by an increase in mortality [2]. Furthermore, impaired cognitive function after critical illness, particularly in patients who suffered delirium, is increasingly being recognised [3]. To date, the exact mechanisms of sepsis-associated delirium, most probably multifactorial in origin, remain obscure. Important precipitating factors possibly include reduced cerebral blood flow (CBF) and oxygen extraction by the brain, disruption of the blood-brain barrier and cerebral oedema that may arise from the action of inflammatory mediators on the cerebrovascular endothelium, abnormal neurotransmitter composition of the reticular activating system, impaired astrocyte function, and neuronal degeneration [4]. As sedation and other treatments often obscure the neurological picture, the diagnosis of delirium in patients with sepsis is difficult. Accordingly, there is considerable variability in reported incidences, ranging from 8% to 70%, which seems to arise at least in part from differences in diagnostic criteria [4]. The term sepsis-associated delirium has recently been proposed to replace the term septic encephalopathy in order to comply with changes in classifications of the *Diagnostic and Statistical Manual of Mental Disorders *(4th edition) and the International Statistical Classification of Diseases and Related Health Problems (ICD-10) [5].

Previous work on cerebral perfusion and cerebrovascular reactivity in sepsis has yielded conflicting results. In a retrospective analysis, hypotension was shown to be the only predictor of delirium in post-operative patients with sepsis [6]. Bowton and colleagues [7] found low CBF in patients with sepsis and these results suggest a role of cerebral ischaemia in the development of sepsis-associated delirium. In contrast, a recent study on cerebral haemodynamics in mechanically ventilated patients with sepsis-associated delirium [8] reported normal global CBF measured with transcranial Doppler (TCD). However, a SPECT (single photon emission computed tomography) study in a small group of general medical patients showed that frontal or parietal cerebral perfusion abnormalities occur in delirium [9]. To date, two studies have been undertaken to address the issue of cerebral autoregulation in patients with sepsis, again yielding inconclusive results. Matta and Stow [10] reported intact pressure autoregulation and cerebral carbon dioxide reactivity in 10 patients with sepsis, whereas Smith and colleagues [11], using carotid TCD and cardiac output measurements, demonstrated that CBF was correlated with cardiac index in septic shock patients, a finding the authors rated as consistent with a loss of cerebrovascular autoregulation. Neither study differentiated between patients with sepsis-associated delirium and those without sepsis-associated delirium.

The role of biomarkers in sepsis-associated delirium is even less clear. Potential markers for delirium have recently been reviewed [12], but much research has focused on patients with delirium independent of sepsis. Furthermore, it is not clear whether the results also apply to patients with sepsis. It would be helpful to have reliable serum markers that support the diagnosis of sepsis-associated delirium. Recent research has investigated the value of S-100β and neuron-specific enolase (NSE) [13,14]. However, the endpoints of these studies were mortality and irreversible brain injury. The results of these two studies are contradictory and difficult to compare due to marked differences between the protocols. Furthermore, sepsis-associated delirium may or may not lead to permanent brain damage [5].

In view of the many questions regarding the pathophysiology of sepsis-associated delirium, we addressed three aspects. Given the CBF data, reduced cerebral perfusion is a possible cause of sepsis-associated delirium. We, therefore, tested the hypothesis that patients with sepsis-associated delirium have alterations in cerebral perfusion. The response of the brain to the intense inflammatory stimulus associated with sepsis is an additional key factor in the development of sepsis-associated delirium. Therefore, we tested the hypothesis that there is an association between sepsis-associated delirium and the inflammatory response reflected by interleukin-6 (IL-6) and C-reactive protein (CRP). Finally, in view of the diagnostic difficulties, we addressed the question of whether S-100β and basal cortisol are potential markers for sepsis-associated delirium.

## Materials and methods

This study was approved by the regional ethics committee. Written informed consent was obtained from all patients or their closest relatives. Patients admitted to the intensive care unit were eligible if they were at least 18 years old and had sepsis, severe sepsis, or septic shock according to the criteria of the 2001 SCCM/ESICM/ACCP/ATS/SIS (Society of Critical Care Medicine/European Society of Intensive Care Medicine/American College of Chest Physicians/American Thoracic Society/Surgical Infection Society) International Sepsis Definitions Conference [15]. Patients with an intracranial focus of infection, with a relevant pre-existing central neurological disorder, or with delirium attributable to a cause other than sepsis were excluded. All patients were studied after stabilisation within 48 hours of admission to the intensive care unit. No interventions were performed in this strictly observational study. Patient management and treatment changes were left entirely to the discretion of the attending physicians.

Sepsis-associated delirium was diagnosed using the confusion assessment method for the intensive care unit (CAM-ICU) [16]. Sedated patients were examined at the end of the routinely performed daily sedation pause. Patients in whom sedation was not stopped were not assessed and were excluded from this study. Patients with possible alcohol withdrawal delirium, acute or chronic hepatic failure, or uncorrected metabolic derangements were excluded. Routine monitoring included electrocardiography, pulse oximetry, and mean arterial pressure (MAP) measured directly in the radial or femoral artery. During the examination, patients were in the supine position with a head elevation of no more than 30°. As a surrogate for cerebral oxygenation, a tissue oxygenation index (TOI) was assessed by near-infrared spectroscopy (NIRS) [17] with measurements performed bilaterally over the frontal to frontoparietal area (NIRO-200; Hamamatsu Photonics K.K., Hamamatsu City, Japan). Using TCD with a 2-MHz probe (Multidop T; DWL, Singen, Germany), blood flow velocity (FV) in the middle cerebral artery of both hemispheres was monitored for 1 hour. Analogue outputs from arterial pressure monitoring and TCD were transferred to a laptop computer via an analogue-to-digital converter and processed using the 'ICM^+ ^software', version 6.1, from the University of Cambridge, UK [18]. Cerebrovascular autoregulation was assessed by calculating a moving correlation coefficient (the index of cerebrovascular autoregulation, Mx) between MAP and FV as described previously [19]. Briefly, values of MAP and FV that are calculated every 10 seconds by the bedside software are used for calculation of the index Mx. Mx is calculated every 60 seconds as the moving linear correlation coefficient between the last 30 consecutive values of MAP and FV. A positive correlation coefficient indicates impaired autoregulation, and a correlation coefficient close to zero or negative indicates intact autoregulation. Values of Mx of greater than 0.3 have been shown to be associated with disturbed autoregulation [20]. For analysis, data from the two hemispheres were averaged and the mean of each parameter over the 60-minute recording period was used for subsequent analyses.

CRP, IL-6, S-100β, and cortisol were determined during each monitoring session. IL-6 was measured using a solid-phase enzyme-labelled chemiluminescent sequential immunometric assay (Immulite 2000 IL-6; Siemens Medical Solutions Diagnostics, Los Angeles, CA, USA). For S-100β, the manufacturer (Roche Diagnostics GmbH, Mannheim, Germany) proposes a cutoff of 0.105 μg/L on a detection range of 0.005 to 39 μg/L for patients with possible cerebral damage (sensitivity 99%, specificity 33%). Cortisol was measured with an Immulite 2000 cortisol assay (Siemens Healthcare Diagnostics, Los Angeles, CA, USA). The reference range for diurnal variation given by the manufacturer is 138 to 690 nmol/L.

A non-parametric approach was used for analysis as data are clearly not normally distributed. Comparisons were made using the Mann-Whitney *U *test. Calculations were performed with SPSS 15.0 for Windows (SPSS Inc., Chicago, IL, USA). Data are shown as median (range) unless specified otherwise. A two-tailed *P *value of less than 0.05 was considered significant.

## Results

Between January and July 2007, 23 consecutive patients were eligible for inclusion and consented to participate. Seven patients had to be excluded from the analysis. One patient developed an acute intracranial pathology manifesting with a unilaterally dilated pupil, coma, and death. In six patients, continuous deep sedation precluded a reliable assessment of delirium with the CAM-ICU. Sepsis-associated delirium was diagnosed in 12 of the remaining 16 patients. The median patient age was 74.5 (18 to 90) years, 38% were female, and the median APACHE II (Acute Physiology and Chronic Health Evaluation II) score at admission was 22.5 (9 to 36). Patients with sepsis-associated delirium had higher median APACHE II scores (23 versus 13) but this difference did not reach statistical significance (*P *= 0.09). Thirty-day mortality was 38%. All patients who died had sepsis-associated delirium. Patient characteristics are shown in Tables 1 and 2. The median Glasgow Coma Scale score was lower in patients with sepsis-associated delirium (11 [5 to 14] versus 15 [11 to 15]; *P *= 0.028). Recombinant activated protein C was not used in this group of patients.

**Table 1:** Patient characteristics I

Patient	Delirium (CAM-ICU criteria)	Gender	Age, years	APACHE II score	Source of sepsis	Causative organism
1	Yes (I, II, III)	Male	55	12	Pneumonia	Unknown
2	Yes (I, II, III, IV)	Male	81	26	Pneumonia	Unknown
3	Yes (I, II, III, IV)	Male	70	16	Pneumonia	*Streptococcus pneumoniae*
4	Yes (I, II, IV)^a^	Male	74	21	Abdominal	Unknown
5	Yes (I, II, IV)^a^	Male	70	23	Pneumonia	*Enterobacter cloacae*
6	Yes (I, II, IV)	Female	75	32	Abdominal	*Escherichia coli*
7	Yes (I, II, IV)^a^	Female	79	23	Pneumonia	*Streptococcus pyogenes*
8	Yes (I, II, IV)^a^	Female	76	26	Prosthetic joint infection	*Staphylococcus aureus*
9	Yes (I, II, IV)^a^	Female	68	22	Abdominal	Unknown
10	Yes (I, II, IV)	Male	83	36	Pneumonia	*Enterobacter aerogenes*
11	Yes (I, II, IV)	Male	85	22	Abdominal	*Bacteroides fragilis*
12	Yes (I, II, IV)^a^	Male	75	31	Abdominal	*Bacteroides fragilis*
13	No	Female	59	27	Pneumonia	Unknown
14	No	Male	52	9	Pneumonia	*Streptococcus pneumoniae*
15	No	Male	90	15	Necrotizing cholecystitis	*Klebsiella oxytoca*
16	No	Female	18	11	Pneumonia	Unknown

Identified criteria of the confusion assessment method for the intensive care unit (CAM-ICU): I, acute onset of changes or fluctuations in the course of mental status; II, inattention; III, disorganized thinking; IV, altered level of consciousness. ^a^Patient died. APACHE II, Acute Physiology and Chronic Health Evaluation II.

**Table 2:** Patient characteristics II

Patient	Time^a^	Intubated	PaO_2_^b^	Glc^c^	Heparin, IU/24 hours	NA^d^	DOB^e^	Steroids^f^	Sedation^g^
1	30		10.2 (8.0)	13.3	10,000				L, H
2	42		13.6 (11.0)	8.3	22,000				L, H, Q
3	39	X	9.2 (7.6)	8.0	10,000			X	M, F
4	37		8.3 (8.3)	6.3	LMWH 5,000			X	P, M
5	30	X	11.3 (9.1)	9.6	15,000	18			P, M
6	39	X	16.2 (9.3)	5.3	10,000	14		X	P, Mi, M
7	29		11.5 (8.4)	6.5	20,000	7	300		None
8	42		8.6 (6.8)	5.3	20,000	11	400		M
9	48	X	13.4 (12.1)	6.9	10,000	26		X	Mi, M
10	46	X	15.0 (10.2)	7.0	LMWH 2,500	20		X	Mi, F, M
11	48	X	11.3 (8.6)	5.7	LMWH 5,000	18		X	P, M, R
12	42		18.5 (15.1)	7.6	15,000				P, M
13	25	X	19.2 (9.3)	4.8	LMWH 5,000				P, F
14	6		13.8 (12.0)	6.7	None			X	None
15	44		11.7 (11.6)	6.1	LMWH 5,000		300		M, H
16	26		11.8 (11.2)	6.8	LMWH 5,000				None

Patients 1 to 12: sepsis-associated delirium present; patients 13 to 16: no sepsis-associated delirium. ^a^Time, time interval (hours) between admission to the intensive care unit and measurements. ^b^PaO_2_, partial pressure of oxygen during measurement (lowest recorded value between admission to the intensive care unit and measurement). ^c^Glc, blood glucose levels (mmol/L). ^d^NA, noradrenaline: μg/minute during measurement. ^e^DOB, dobutamine: μg/minute during measurement. ^f^Patient 3: 3 × 100 mg hydrocortisone per 24 hours; patient 14: 25 mg methylprednisolone per day; all other patients: 4 × 50 mg hydrocortisone per 24 hours. ^g^F, fentanyl; H, haloperidol; L, lorazepam; M, morphine; Mi, midazolam; P, propofol; Q, quetiapine; R, remifentanil. LMWH, low-molecular-weight heparin.

**Figure 1:**
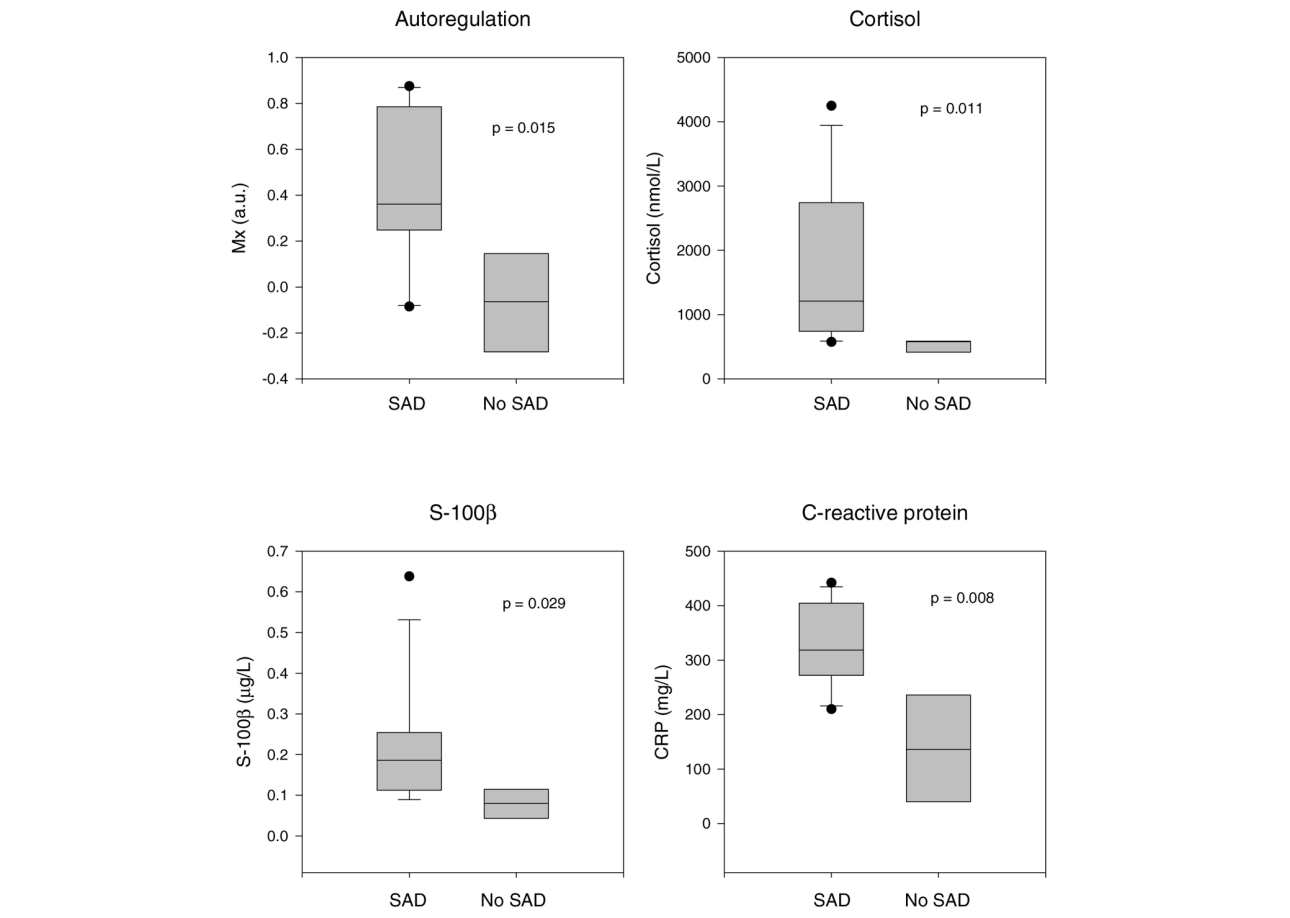
Autoregulation, C-reactive protein (CRP), S-100β, and cortisol are significantly different in patients with and without sepsis-associated delirium (SAD). a.u., arbitrary units; Mx, index of cerebrovascular autoregulation.

Haemodynamic, respiratory, and cerebral perfusion data are shown in Table 3. Seven patients, all of whom had sepsis-associated delirium, required noradrenaline for haemodynamic support. There was no significant difference in MAP or cerebral perfusion assessed with TCD and NIRS in the two groups of patients. However, the calculated index of autoregulation was significantly different between these groups (*P *= 0.015) (Figure 1). There were no significant correlations between Mx, the index of autoregulation, and APACHE II score or Mx and catecholamine requirements.

**Table 3:** Haemodynamics, cerebral perfusion, and respiratory parameters

	Sepsis-associated delirium	No sepsis-associated delirium	*P *value
Mean arterial pressure, mm Hg	75 (57–87)	85 (73–94)	0.1
FV, cm/second	76 (40–97)	48 (45–98)	0.3
Cerebral TOI, percentage	59 (49–74)	65 (59–69)	0.2
SaO_2_, percentage	97 (91–100)	99 (93–100)	0.2
PaCO_2_, kPa	5.4 (3.7–9.4)	5.3 (4.5–5.5)	0.7
Ear temperature, °C	37.1 (35.0–38.6)	37.3 (36.3–38.5)	0.5

All values are shown as median (range) and represent means of data collected during a 60-minute measurement. FV, cerebral blood flow velocity in the middle cerebral artery; PaCO_2_, arterial partial pressure of carbon dioxide; SaO_2_, arterial oxygen saturation; TOI, tissue oxygenation index. TOI and FV are averaged values from both cerebral hemispheres. *P *values were calculated with the Mann-Whitney *U *test.

**Figure 2:**
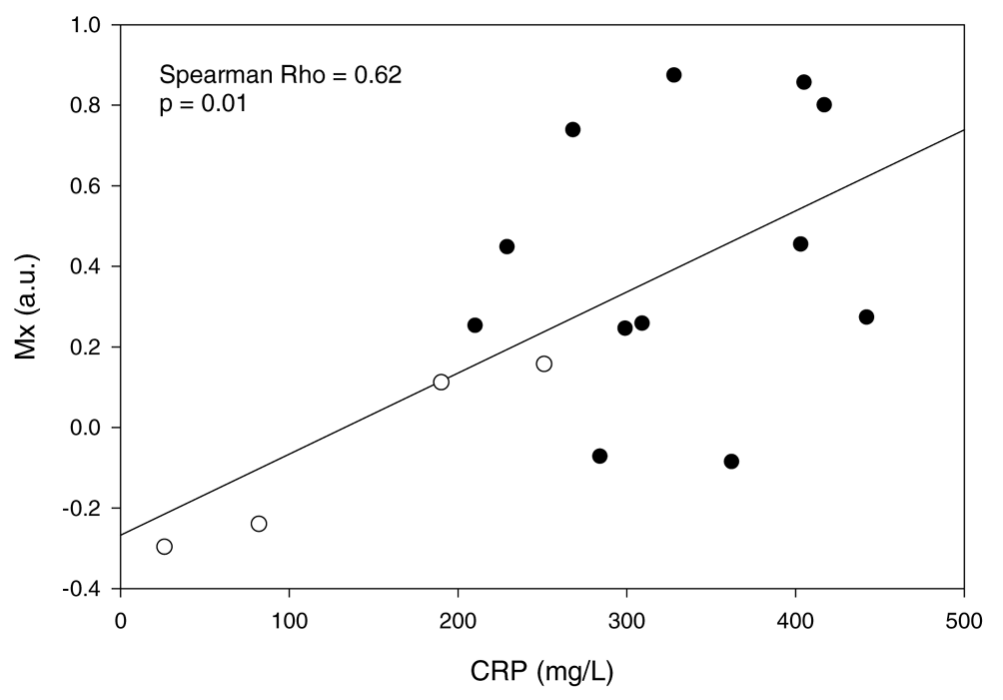
Higher values of C-reactive protein (CRP) are significantly correlated with increasingly disturbed autoregulation. Open circles represent patients without sepsis-associated delirium and black circles represent patients with sepsis-associated delirium. a.u., arbitrary units; Mx, index of cerebrovascular autoregulation.

Patients with sepsis-associated delirium had higher CRP levels (*P *= 0.008) (Figure 1). In contrast, no significant differences were found for IL-6 levels (378 [21 to 8,299] versus 86 [42 to 1,117] pg/mL; *P *= 0.3) in patients with and without sepsis-associated delirium, respectively. Interestingly, higher CRP levels were correlated with increasingly disturbed autoregulation (Spearman rho = 0.621, *P *= 0.01) (Figure 2).

With regard to possible serum markers, we found significant associations with sepsis-associated delirium for both S-100β (*P *= 0.029) and cortisol (*P *= 0.011) (Figure 1). S-100β, but not cortisol, discriminated between survivors and non-survivors (0.103 [0.036 to 0.193] and 0.247 [0.153 to 0.638] μg/L, respectively; *P *= 0.003).

## Discussion

In our small group of patients, cerebral perfusion assessed with TCD and NIRS did not differ between patients with and without sepsis-associated delirium. However, the state of autoregulation differed between the two groups. The correlation between CRP and Mx suggests that this may be due to inflammation impeding cerebrovascular endothelial function. The potential delirium markers S-100β and cortisol were different in patients with and without sepsis-associated delirium.

The concept of inadequate cerebral perfusion as one contributor to brain damage in sepsis is supported by earlier work showing reduced CBF in patients with sepsis by means of the xenon-133 clearance technique [7]. Wijdicks and Stevens [6], though in a retrospective design, found severe hypotension to be the only predictor of sepsis-associated delirium in a multiple logistic regression analysis. In our patients, MAP was a therapeutic target and was tightly controlled, which may explain why we did not find an association between MAP and sepsis-associated delirium. A recent study, also using TCD, found normal FV in patients with sepsis-associated delirium [8]. In our patients, the results of the TCD measurements were highly variable (Table 3). In our opinion, it is not possible to define a normal range of FV in such a group of patients. Differences in patient age, sedation, arterial partial pressure of carbon dioxide (PaCO_2_), and other factors will influence not only CBF but also the relationship between CBF and FV. It is, therefore, impossible to draw conclusions on absolute CBF between the groups of patients with and without sepsis-associated delirium on the basis of a single 'snapshot' measurement of FV.

NIRS is an increasingly used non-invasive tool to assess cerebral oxygenation. The TOI has been satisfactorily validated [17], and recent work has confirmed that it is not influenced by external factors such as haemoglobin concentration or skull thickness [21]. We did not find conclusive differences in TOI in our patients. There are at least three possible explanations for this. First, disseminated small hypoxic areas or leucoencephalopathic lesions, as documented in a recent magnetic resonance imaging (MRI) study of nine patients with septic shock [22], are probably too small to be detected by NIRS. Second, we placed the NIRS optodes over the frontal to frontoparietal region. While a SPECT study in medical patients with delirium found regional CBF changes in these areas [9], it is possible that these areas are not very susceptible to ischaemia in sepsis-associated delirium. Lower brain structures such as basal ganglia and the thalamus might be more important in the development of sepsis-associated delirium. In a case report of a patient with severe sepsis-associated delirium, MRI demonstrated abnormalities in the midbrain, vermis of the cerebellum, and medial portions of both temporal lobes. Extensive infarction of the basal ganglia was revealed at the autopsy of this patient [23]. Another explanation could be that brain ischaemia, though suggestive, is not the only cause of neuronal damage in sepsis-associated delirium. Apoptotic neuronal death in sepsis has been reported by several authors [24,25] and it has been suggested that this is triggered by the pro-inflammatory mediator nitric oxide rather than by ischaemia [26].

To date, two studies have investigated cerebral autoregulation in patients with sepsis, yielding inconclusive results [10,11]. Our results suggest that sepsis-associated delirium, but not sepsis *per se*, is associated with impaired pressure autoregulation. Cerebrovascular autoregulation is dependent on cerebral endothelial function, and endothelial dysfunction is a key feature in sepsis. One of its characteristics is an inhibition of vasodilatation [27]. If this also occurred in the cerebral circulation, it could explain autoregulatory failure. Currently, there are only few data on cerebral endothelial dysfunction in sepsis. Cerebral perivascular oedema, another possible consequence of endothelial dysfunction, has been described in animal models by several authors [24,25,28]. If the cerebrovascular endothelium is affected to a relevant degree, this could potentially have implications for therapy. Perhaps, high cerebral perfusion pressures should be avoided in order to decrease oedema formation. The significant correlation between Mx and CRP does not imply a causal relationship between inflammation and autoregulation. However, one could speculate that disturbance of autoregulation may be the result of the inflammatory response. An association between IL-6 and autoregulation would have supported this concept. However, such a relationship was not found in our patients. This may be explained by the fact that fluctuations of IL-6 occur much more rapidly than CRP levels or that changes in autoregulatory status have a different temporal pattern than changes in IL-6. However, further investigations into the relationship between inflammation and cerebrovascular function are warranted. It would be valuable if, for example, monitoring of autoregulation could be used to quantify the effects of an inflammatory insult to the brain.

In our patients, elevated CRP, S-100β, and cortisol were associated with sepsis-associated delirium. The association between CRP and delirium has been described previously in non-septic patients [29]. With regard to S-100β, our data are consistent with those from patients with delirium after cardiac surgery [30]. Is this increase in S-100β due to brain injury? The interpretation of S-100β, a protein found predominately in astrocytes and Schwann cells, is difficult. Even when an increase in S-100β is not due to extracranial sources, including the heart, skeletal muscle, and kidneys [31], it is not absolutely specific for brain damage [32] but may also indicate a disturbance of the blood-brain barrier [33]. It has been suggested that low values reflect blood-brain barrier dysfunction, whereas higher values reflect brain damage. A cutoff value has been suggested based on a pharmacokinetic model [34]. However, S-100β cutoff values depend on the kit used, and comparisons can be made only when identical kits have been used. In our patients, we found moderate elevations of S-100β, but we cannot differentiate between blood-brain barrier dysfunction and glial or neuronal damage. Some of our patients had acute renal failure and haemofiltration, but neither renal failure [13] nor haemofiltration [35] influences S-100β levels. We did not measure NSE, another possible marker of brain damage. However, in a large study including 170 patients with severe sepsis and septic shock, a similar proportion of patients showed increased S-100β and NSE levels, with S-100β being a better predictor of disease severity [13].

Elevated cortisol levels have been associated with delirium in Cushing syndrome and high-dose steroid treatment [12]. However, to our knowledge, there are only two small studies investigating cortisol as a marker for delirium in general medical or surgical patients [36,37]. A further study suggested that patients who fail to suppress their cortisol production after a suppression test with dexamethasone are at increased risk for delirium [38]. While this view is interesting, there are several important issues that preclude our finding from supporting the hypothesis that cortisol is a useful marker of sepsis-associated delirium. First, high cortisol levels may simply be an indicator of a high degree of the systemic inflammatory response (that is, an indicator of more severe disease) [39]. Second, some of our patients had hydrocortisone therapy (Table 2), again possibly reflecting more severe disease. Accordingly, the association between high cortisol levels and sepsis-associated delirium would reflect severity of disease rather than a direct relationship. It is plausible that patients with more severe sepsis are at higher risk of developing sepsis-associated delirium. Despite the fact that we did not find a significant association between sepsis-associated delirium and APACHE II score, others reported such a relationship [40]. Finally, a further concern is related to the method of measurement. It was recently shown that immunoassay estimation of total plasma cortisol in septic patients, as performed in our study, shows wide assay-related variation [41].

There are several limitations to the present study. First, the number of investigated patients is small. Therefore, these preliminary results need to be confirmed in a larger group of patients. We could not control PaCO_2 _in this group of patients. Performing measurements at standardised PaCO_2 _levels was not feasible in this observational study as a relevant number of our patients either were breathing spontaneously or, if intubated, had a ventilator-assisted form of spontaneous breathing. While PaCO_2 _was stable during measurements, it is a key denominator of CBF and cerebrovascular autoregulation. This aspect is further complicated by the conflicting data on cerebrovascular CO_2 _reactivity in sepsis. A recent study found normal CO_2 _reactivity in 10 mechanically ventilated patients with sepsis-associated delirium [8]. This is supported by earlier work by Bowton and colleagues [7] and Matta and Stow [10]. However, Terborg and colleagues [42] found impaired CO_2 _reactivity, and Bowie and colleagues [43] reported values ranging from reduced to exaggerated CO_2 _responses. Autoregulation is also influenced by temperature [44], and again we could not control for this parameter. However, the range of temperatures at which we performed our measurements was moderate (Table 2).

## Conclusion

In this small group of patients, cerebral perfusion assessed with TCD and NIRS did not differ between patients with and without sepsis-associated delirium. However, the state of cerebrovascular autoregulation differed significantly between the two groups. This may be due to inflammation impeding cerebrovascular endothelial function, a concept that is supported by the significant correlation between elevated CRP and disturbed autoregulation. Further investigations defining the role of S-100β and cortisol as aids in the diagnosis of sepsis-associated delirium are warranted.

## Key messages

• In this small group of patients, cerebral perfusion assessed with transcranial Doppler and near-infrared spectroscopy did not differ between patients with and without sepsis-associated delirium.

• We found a significant association between disturbed cerebrovascular autoregulation and sepsis-associated delirium.

• A significant correlation between higher values of C-reactive protein and increasingly disturbed cerebrovascular autoregulation suggests a harmful effect of inflammation on cerebrovascular endothelial function.

• The significant associations between sepsis-associated delirium and elevated S-100β and cortisol suggest that further investigations defining the role of these markers as aids in the diagnosis of sepsis-associated delirium are warranted.

## Abbreviations

APACHE II = Acute Physiology and Chronic Health Evaluation II; CAM-ICU = confusion assessment method for the intensive care unit; CBF = cerebral blood flow; CRP = C-reactive protein; FV = flow velocity; IL-6 = interleukin-6; MAP = mean arterial pressure; MRI = magnetic resonance imaging; Mx = index of cerebrovascular autoregulation; NIRS = near-infrared spectroscopy; NSE = neuron-specific enolase; PaCO_2 _= arterial partial pressure of carbon dioxide; SPECT = single photon emission computed tomography; TCD = transcranial Doppler; TOI = tissue oxygenation index.

## Competing interests

The authors declare that they have no competing interests.

## Authors' contributions

DP carried out the data collection and analysis and drafted the manuscript. MS, SCUM, and HP participated in the study design and critically revised the manuscript for important intellectual content. SD-K performed data and statistical analysis and critically revised the manuscript for important intellectual content. PS adapted the ICM^+ ^software to our specific needs and performed data quality control. SR participated in the study design, data collection, and analysis. SPS participated in the study design, acquired funding, and critically revised the manuscript for important intellectual content. LAS developed the study concept, supervised data collection and analysis, acquired funding, and drafted and revised the manuscript. All authors read and approved the final manuscript.
